# Virtual reality for the promotion of interoception awareness and body image in breast cancer survivors: a study protocol

**DOI:** 10.3389/fpsyg.2023.1165905

**Published:** 2023-06-01

**Authors:** Valeria Sebri, Ilaria Durosini, Milija Strika, Silvia Francesca Maria Pizzoli, Ketti Mazzocco, Gabriella Pravettoni

**Affiliations:** ^1^Applied Research Division for Cognitive and Psychological Science, IEO, European Institute of Oncology IRCCS, Milan, Italy; ^2^Department of Oncology and Hemato-Oncology, University of Milan, Milan, Italy; ^3^Faculty of Psychology, Università Cattolica del Sacro Cuore, Milan, Italy

**Keywords:** virtual reality and interoception breast cancer survivors, body image, virtual reality, interoception, wellbeing

## Abstract

Women who received a diagnosis of breast cancer often report impairments in physical and psychological wellbeing, even some years after treatments. Individual awareness about physical changes, body image, and current sensations related to their body is important to maintain a psycho-emotional balance. Virtual reality, as an advanced human–computer interface, can be an effective tool to improve breast cancer survivors' abilities to know and manage their current sensations related to their bodies. The present study protocol proposes a virtual reality intervention aiming at promoting interoception and emotional wellbeing, fear of cancer recurrence, and body perception in breast cancer survivors, according to the three data collection times. Repeated-measure analysis of variance (ANOVA) with between–within interaction will be performed. Expected results include participants' awareness of their internal feelings, the reduction of negative emotions, and the management of symptoms related to the body, clarifying characteristics for the effective implementation of VR psychological intervention in the future.

## Introduction

Body image (BI) is defined as the internal representation of one's outward appearance (Thompson et al., 1999), also including its related emotions and thoughts (Lewis-Smith et al., 2018; Sebri et al., 2020). BI involves perceptual (e.g., accuracy in estimating one's body), affective (feelings and emotions), attitudinal (the degree of satisfaction related to one's body), cognitive (beliefs and thoughts), and behavioral (e.g., possible compensatory behaviors enacted to achieve a satisfactory self-image) levels (Cash and Smolak, 2011). Considering the malleability of the self [It is conceptualized as a function of social and cognitive contexts demonstrating that mood can lead to temporary changes in the self (Markus and Kunda, 1986; Markus and Nurius, 1986).] and the motivation dynamics after cancer (Durosini et al., 2021), significant and/or traumatic events (such as an oncological experience) can affect BI strongly (Sebri et al., 2020). The literature highlights that women with previous breast cancer experience may live with negative emotions that affect their quality of life (Durosini et al., 2022) and develop a negative BI due to physical and psychological issues after diagnosis and oncological treatment and interventions (Maass et al., 2015; Sherman et al., 2018). In BI evaluation, inner sensations and related awareness play a relevant role. Particularly, breast cancer survivors may perceive bodily sensations never felt before, increasing interoceptive awareness, especially about the breast(s) (Paterson et al., 2016). As a definition, interoception refers to the ability to be aware of one's internal sensations, such as itching and hunger. The debate about the role of interoception awareness and its impact on emotions is ongoing. On the one hand, literature has stated that such awareness is important to regulate internal states and maintain a psycho-emotional balance (Herbert and Pollatos, 2012), promoting emotional regulation and positive overall self-representation and self-appraisals. On the other hand, being aware of interoception and its related inner sensation could increase the focus on internal sensations as it is linked to the fear of cancer recurrence. Controlling behaviors, such as constantly looking for breast lumps, may frequently emerge increasing negative emotions, such as distress and anxiety (Humphris and Ozakinci, 2008). Thus, effective psychological intervention to promote an effective awareness of the interplay between internal and external sensations is crucial to increase recognition and acceptance of the body and its related inner sensations after illness, without increasing negative emotions (Raimo et al., 2021; Sebri et al., 2022). In other words, increasing interoceptive capacity can, therefore, support women in recognizing their feelings and regulating related emotions (Reed et al., 2016; Czamanski-Cohen et al., 2019). Additionally, following Higgins' discrepancy theory (Higgins et al., 1985), the cognitive dissonance between current and ideal self-images could induce concerns and discomfort related to BI, with important consequences on emotions (anxiety and depression in particular), quality of life, and social relationships. Accordingly, the perception of the stigma as a “patient” referring to illness characteristics could decrease individuals' empowerment, improving shame and social isolation (Amini-Tehrani et al., 2021; Brunet and Price, 2021).

Virtual reality (VR), as a tool that allows users to experience a sense of belonging with a body in a virtual world (*virtual reality full body illusion*), is generally involved in promoting body awareness and regulation of emotional wellbeing, especially in the eating disorder fields (Slater et al., 2008; Serino et al., 2016; So et al., 2022). Starting with the process of embodiment and the sense of presence, users can, therefore, perceive their real and virtual bodies simultaneously (Ventura et al., 2018). Such an experience of the virtual body belonging is the result of the combination of visual and tactile stimulations and individuals' ability to identify their own body (*self-identification*) that occupies a specific space (*self-location*) and its related internal sensations (Haugstad et al., 2006; Moussally et al., 2017; Nakul et al., 2020).

In accordance with the present theoretical framework, it might be interesting to investigate the VR application aiming at improving BI awareness and wellbeing in women who received a cancer diagnosis. The overall purpose of this research protocol was to implement a VR intervention to improve interoceptive ability and emotional wellbeing in breast cancer survivors. Specifically, the objectives of the present protocol study are to evaluate the impact of the following:

- Sensation manipulation to promote interoception awareness and psychological wellbeing, positive self-appraisals, and emotional regulation (reducing, for example, the fear of cancer recurrence) through VR;
- A VR intervention on body awareness and negative body sensations following illness, addressing interoceptive sensations related to the body.

We expect that the present study focusing on VR intervention may help women with a history of breast cancer to improve their psychological wellbeing and awareness of inner sensations, promoting better emotional regulation.

## Methods

### Participants

Women who have experienced breast cancer in the past and that have completed cancer treatments will be included in this study. Specifically, inclusion criteria are as follows: (a) adult women (18 years and older); (b) women who have previously received a diagnosis of breast cancer (stage I and II); (c) women who have received cancer treatment (e.g., chemotherapy, radiotherapy, or monotherapy) in the past. Contrarily, exclusion criteria include people with a diagnosis of metastatic cancer and women who are unable to sign an informed consent and/or have poor knowledge of the Italian language. A sample size of at least 40 participants (please see “Data Analysis and Sample Size Estimation” section) is estimated to be involved.

### Procedure and measures

The study duration will be ~12 months, from recruitment until the conclusion of T2. Researchers will contact various breast cancer patient associations to share the current project and promote contact with interested people. Women interested in participating in the present research project can contact the researchers by writing to the email address provided by the association. The study will also be publicized through social networks (e.g., Facebook, LinkedIn, and Instagram), inviting interested women to contact the researchers through the email indicated in the study announcement. No monetary compensation will be provided, and mandatory adherence to the study will be requested as informed consent before administering the questionnaires. It will be made clear that participation in the research is completely free, voluntary, and free of charge. All participants will have the option to discontinue the research at any time without providing any explanation. At the end of the VR intervention, participants will be asked for their willingness to provide a personal contact that will be exclusively used to send them the follow-up questionnaires after 1 month (T2). Regarding the questionnaires, a code will be assigned to make the collected data pseudonymized.

The study will be conducted following the Declaration of Helsinki principle and informed written consent will be collected from all participants. The study protocol has been revised and approved by the Ethical Committee of the University of Milan.

Before VR intervention, participants will be randomized into the two research groups through Excel software (RAND function). Women in the experimental group will be invited to wear a 3D oculus and follow specific steps of the present intervention. Specifically, women will be shown a female avatar. The proposed virtual environment will be located in a neutral room, and the avatar will have an average body size compared to the general population. Women will be able to see the avatar's entire body looking straight ahead through the use of a 3D oculus. Then, visual and auditory stimuli will allow for an increased sense of belonging with respect to the virtual body, as described by Slater et al. (2008). To sum up, the intervention will consist of viewing a silhouette of a body through VR and following a series of visual and auditory stimuli to improve their abilities to perceive and control internal sensations through VR, whereas participants included in the control group will be asked to wear a 3D oculus for VR viewing. Unlike the previous group, participants will be invited to observe an immersive experience for relaxation purposes as a neutral stimulus. The relaxation experience related to the control group will last the same time as the intervention experience for the experimental group. Breast cancer survivors who will belong to the control group will not receive different instructions in terms of VR characteristics and how to use it.

More specifically, the proposed intervention will be structured as follows:

#### Phase 1 (T0)

Following the presentation of the study and the signing of the informed consent, participants will be asked to fill out the socio-demographic data, the clinical history of illness, any ongoing psychological support course, and a series of questionnaires aimed to explore some psychological aspects. Specifically, the following questionnaire will be administered:

- *The multidimensional assessment of interoceptive awareness* (MAIA; Mehling et al., 2012): The MAIA is a self-report questionnaire that assesses eight dimensions related to interoceptive aspects of body awareness. The scale has a total of 32 items on a 6-point Likert scale, from 0 (never) to 5 (always), and covers eight “distinct but related” dimensions of interoception: *awareness of bodily sensations* (e.g., “I can tell where I feel good in my body”), the *tendency to ignore uncomfortable bodily sensations* (e.g., “I distract myself when I feel uncomfortable or fearful sensations”), the *ability to have emotional reactions following negative sensations* (e.g., “I worry if I feel pain or uncomfortable sensations”), the *ability to adjust attention regarding multiple sensations* (e.g., “I can focus sensations on my body, even when there are many distractions around me”), the *ability to be aware of the relationship between body states and affective states* (e.g., “I can feel changes in my body when I am happy”), the *ability to pay attention to body states to regulate psychological* distress (e.g., “I can use my breath to help me stay calm and relaxed”), the *ability to feel one's bodily sensations to make decisions* (e.g., “I listen to my body to help me choose what to do”), and *the experience of one's body as safe and trustworthy* (e.g., “I feel that my body is a safe place”). Some subscales measure direct experience with the body, and others are associated with the assessment of cognitive processes, such as self-regulation (Mehling et al., 2012).
- *Cancer Worry Scale* (CWS; Custers et al., 2014; Chirico et al., 2022): The CWS is a self-report questionnaire designed to measure concerns with respect to the recurrence of cancer disease. The questionnaire has eight items on a 4-point Likert scale (from “never” to “almost always”). The higher the score, the greater the concern related to the fear of cancer recurrence.
- *State-Trait Anxiety Inventory* (STAI-Y1; Spielberger, 1983; Spielberger et al., 1983): The STAI is a self-report questionnaire aimed at assessing anxiety as a transient emotional response involving negative feelings related to, for example, nervousness, worry, and tension. As a revised version of the original STAI-X, it presents 20 items on a 4-point Likert scale (from “nothing” to “very much”) with a range of total scores between 20 and 80 (high anxiety).
- *Self-Assessment Manikin* (SAM; Bradley and Lang, 1994): The SAM is a non-verbal self-report measure in which nine figures (manikins) are shown in groups of three to measure the emotional response (positive to negative) and the relative level of activation (high to low) and control (low to high) identified by the subject as central to a specific stimulus.
- *Checking Behaviors*: Some *ad hoc* questions will be administered to measure the frequency and concern of attitudes toward one's internal body sensations. Participants will have to answer some questions on a 10-point Likert scale, including the following: (a) *In the past 7 days, how often have you checked your breasts for lumps?* (b) *In the past 7 days, have you felt anxious?* and (c) *How many times have you told your family members about your fears related to the disease in the past 7 days?*

The duration of T0 will be about 20 min.

#### Phase 2 (T1)

Subsequently, participants will be randomly divided into the experimental and control groups. Participants in the experimental group will be asked to sit on a chair and place their open palms on the table without crossing their legs and keeping their backs straight. Wearing 3D oculus and donning the sensor, the participants will be immersed in VR where they will see the virtual body of an avatar positioned in the same position as them. The experiment will begin with an initial warm-up phase in which participants will be allowed to stand in the virtual environment without receiving additional stimuli (2 min). In this first phase, psycho-physiological indices (heart rate and heart rate variability) will be measured using wearable sensors at the wrist so as not to interfere with the session. Next, the experimental group will observe via VR visual stimulation on different parts of the virtual body (breasts, fingers, and toes) and listen to vocal stimulation, in a consecutive and randomized manner among the group participants. Specifically, this intervention will be structured as follows: The participants will see a light on their virtual body that, starting from an iridescent red color, will fade slowly, becoming white. At the same time, an external voice will guide the participants toward a gradual decrease in sensations of discomfort and itching, in a combination of the visual stimuli. This treatment will initially be proposed for a total of 5 min, considering that 90 s is sufficient to induce body illusion. This process will be proposed in the same mode (duration equal to 5 min) for the body parts of interest of the same (breasts, fingers, and toes), with a 2-min break between stimulations. During the breaks, participants will remain immersed in VR, without additional stimulation. In contrast, participants assigned to the control group, once immersed in a VR depicting a natural environment, will hear only a guiding voice reading a script aimed at relaxation. Once the experiment is over, all participants will fill out the battery of post-intervention questionnaires, which includes the following:

- *The multidimensional assessment of interoceptive awareness* (MAIA);
- *Measures regarding satisfaction related to the intervention*: participants will answer some *ad hoc* questions designed to measure their satisfaction with the intervention;
- *ITC-Sense of Presence Inventory* (ITC-SOPI; Lessiter et al., 2001): The ITC-SOPI is a validated questionnaire consisting of 44 items to explore users' experience of virtual reality. Specifically, it assesses the degree to which subjects experience a “sense of presence in a virtual environment,” how distant the virtual environment is from reality, and how far the virtual environment can be considered a “place.” The items refer to the following four categories: (1) *Spatial Presence*: how physically present users perceive themselves to be in the virtual environment; (2) *Involvement*: how much users perceive the content proposed by the virtual environment; (3) *Ecological Validity*: the level of realism and naturalness of the virtual environment; and (4) *Negative Effects*: the disruptive effects on the physical plane, such as nausea and eye discomfort, that users might experience while immersed in the virtual environment.

#### Phase 3 (T2)

**Figure 1:**
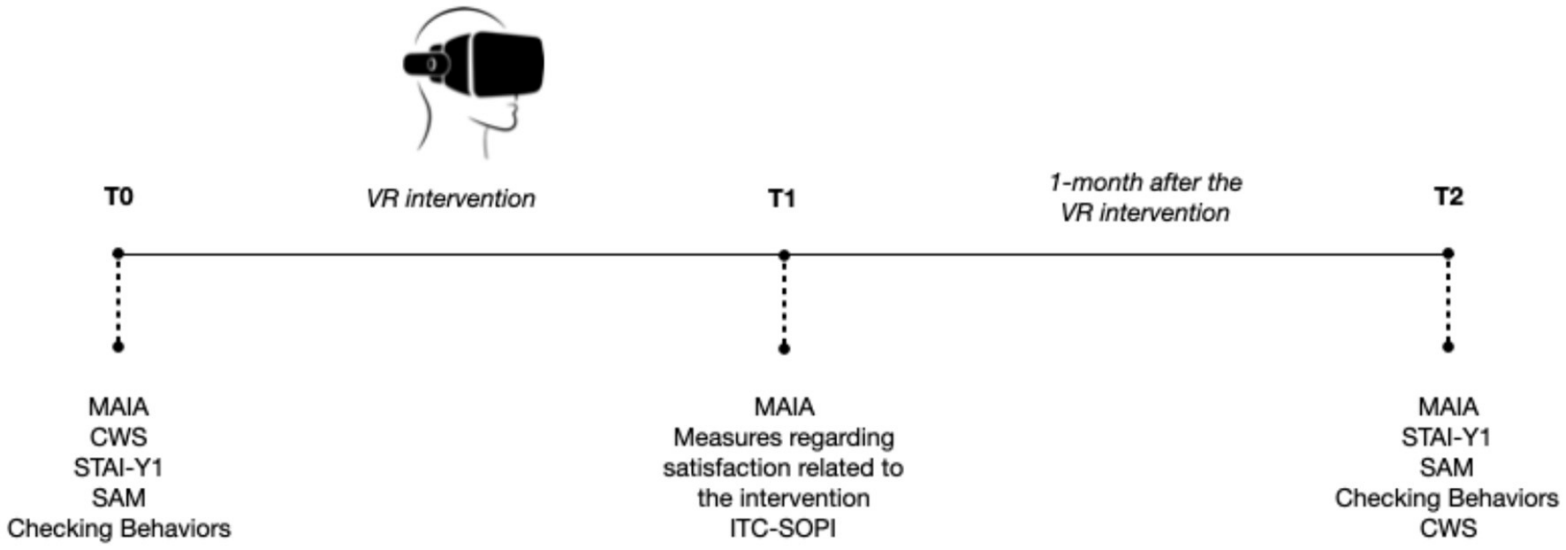
Study procedure.

Lastly, a final administration of questionnaires will be conducted 1 month after the end of the intervention. Specifically, in this phase, the *Multidimensional Assessment of Interoceptive Awareness* (MAIA), the *State-Trait Anxiety Inventory* (STAI-Y1), the *Self-Assessment Manikin* (SAM), and the *Checking behaviors*, the *Cancer Worry Scale* (CWS) will be administered to participants (see Figure 1).

## Data analysis and sample size estimation

An *a priori* estimation of the sample size required for the present study was calculated using G*Power 3.1.9.2 software (Faul et al., 2007) to analyze variance with repeated measure (ANOVA) with between–within interaction. The primary endpoint will be the difference between the two experimental groups (i.e., between-subject factor) in the improvement of the overall MAIA scale score at pre- (T0), post-intervention (T1), and follow-up (T2) times. To detect a weak-median effect size (i.e., partial η^2^ = 0.04), the required sample size is 40 (i.e., 20 participants in each group). The type-I error rate (α) was set at 0.05 (two-sided), and the power (1 – β) was set at 0.90. The collected data could be analyzed using a statistical analysis software, the Statistical Package for Social Science (SPSS, version 27.0).

## Limitations and future research

This study could help people with a history of breast cancer to become more aware of their internal feelings, promoting the reduction of negative emotions (e.g., anxiety) in the long term. Additionally, VR intervention focused on inners sensation awareness and emotional wellbeing may improve the control of possible symptoms (e.g., decreasing the number of breast lump control if it has excessive frequency), reducing anxiety and shame to foster better social relationships (Savioni et al., 2022).

A possible study limitation could be the lack of a state-emotional assessment of breast cancer survivors. Future research needs to assess the state shame and guilt related to the worst memory focused on BI (i.e., State Shame and Guilt Scale—SSGS-8; Cavalera et al., 2017, 2023) of the participants. This could be relevant to monitor differences in negative emotions apart from fear of cancer disease recurrence. Furthermore, including patients under psychotherapy might partially shape the results and the impact of the MBSR protocol.

In conclusion, in this study, there are no anticipated discomforts or undesirable effects for participants. It will be the researchers' responsibility to inform of the possibility for participants to discontinue participation in the research at any time, without giving any explanation and without incurring any possible negative consequences.

## Ethics statement

The Ethics Committee of University of Milan approved the study and all participants signed the informed consent form. This study followed the Declaration of Helsinki.

## Author contributions

VS, ID, MS, and SP designed the study and wrote the protocol. KM and GP revised the draft of the paper. All authors contributed to the article and approved the submitted version.

## References

[B1] Amini-Tehrani M. Zamanian H. Daryaafzoon M. Andikolaei S. Mohebbi M. Imani A. . (2021). Body image, internalized stigma and enacted stigma predict psychological distress in women with breast cancer: a serial mediation model. J. Adv. Nurs. 77, 3412–3423. 10.1111/jan.14881 33969915

[B2] Bradley M. M. Lang P. J. (1994). Measuring emotion: the self-assessment manikin and the semantic differential. J. Behav. Ther. Exp. Psychiatry 25, 49–59. 10.1016/0005-7916(94)90063-9 7962581

[B3] Brunet J. Price J. (2021). A scoping review of measures used to assess body image in women with breast cancer. Psycho Oncol. 30, 669–680. 10.1002/pon.5619 33480160

[B4] Cash T. F. Smolak L. (Eds.). (2011). Body Image: A Handbook of Science, Practice, and Prevention. Guilford press.

[B5] Cavalera C. Pepe A. Zurloni V. Diana B. Realdon O. Jiang R. (2017). A short version of the State Shame and Guilt Scale (SSGS-8). TPM–Testing Psychometr. Methodol. Appl. Psychol. 24, 99–106.

[B6] Cavalera C. Quiroga A. Oasi O. (2023). Ashamed or afraid? Traumatic symptom severity and emotional activations of Covid-19-related events. Asian J. Psychiatr. 82, 103500. 10.1016/j.ajp.2023.103500 36796219 PMC9898053

[B7] Chirico A. Vizza D. Valente M. Iacono M. L. Campagna M. R. Palombi T. . (2022). Assessing the fear of recurrence using the Cancer Worry Scale in a sample of Italian breast cancer survivors. Support. Care Cancer 30, 2829–2837. 10.1007/s00520-021-06718-4 34845503

[B8] Custers J. A. van den Berg S. W. van Laarhoven H. W. Bleiker E. M. Gielissen M. F. Prins J. B. (2014). The Cancer Worry Scale: detecting fear of recurrence in breast cancer survivors. Cancer Nurs. 37, E44–E50. 10.1097/NCC.0b013e3182813a17 23448956

[B9] Czamanski-Cohen J. Wiley J. F. Sela N. Caspi O. Weihs K. (2019). The role of emotional processing in art therapy (REPAT) for breast cancer patients. J. Psychosoc. Oncol. 37, 586–598. 10.1080/07347332.2019.1590491 30929590

[B10] Durosini I. Savioni L. Triberti S. Guiddi P. Pravettoni G. (2021). The motivation journey: a grounded theory study on female cancer survivors' experience of a psychological intervention for quality of life. Int. J. Environ. Res. Public Health 18, 950. 10.3390/ijerph18030950 33499109 PMC7908434

[B11] Durosini I. Triberti S. Savioni L. Sebri V. Pravettoni G. (2022). The role of emotion-related abilities in the quality of life of breast cancer survivors: a systematic review. Int. J. Environ. Res. Public Health 19, 12704. 10.3390/ijerph191912704 36232004 PMC9566755

[B12] Faul F. Erdfelder E. Lang A. G. Buchner A. (2007). G^*^ Power 3: a flexible statistical power analysis program for the social, behavioral, and biomedical sciences. Behav. Res. Methods 39, 175–191. 10.3758/BF03193146 17695343

[B13] Haugstad G. K. Haugstad T. S. Kirste U. M. Leganger S. Wojniusz S. Klemmetsen I. . (2006). Posture, movement patterns, and body awareness in women with chronic pelvic pain. J. Psychosom. Res. 61, 637–644. 10.1016/j.jpsychores.2006.05.003 17084141

[B14] Herbert B. M. Pollatos O. (2012). The body in the mind: on the relationship between interoception and embodiment. Top. Cogn. Sci. 4, 692–704. 10.1111/j.1756-8765.2012.01189.x 22389201

[B15] Higgins E. T. Klein R. Strauman T. (1985). Self-concept discrepancy theory: a psychological model for distinguishing among different aspects of depression and anxiety. Soc. Cogn. 3, 51–76. 10.1521/soco.1985.3.1.51

[B16] Humphris G. Ozakinci G. (2008). The AFTER intervention: a structured psychological approach to reduce fears of recurrence in patients with head and neck cancer. Br. J. Health Psychol. 13, 223–230. 10.1348/135910708X283751 18492319

[B17] Lessiter J. Freeman J. Keogh E. Davidoff J. (2001). A cross-media presence questionnaire: the ITC-Sense of Presence Inventory. Presence Teleoper. Virtual Environ. 10, 282–297. 10.1162/105474601300343612

[B18] Lewis-Smith H. Diedrichs P. C. Harcourt D. (2018). A pilot study of a body image intervention for breast cancer survivors. Body Image 27, 21–31. 10.1016/j.bodyim.2018.08.006 30121489

[B19] Maass S. W. Roorda C. Berendsen A. J. Verhaak P. F. de Bock G. H. (2015). The prevalence of long-term symptoms of depression and anxiety after breast cancer treatment: a systematic review. Maturitas 82, 100–108. 10.1016/j.maturitas.2015.04.010 25998574

[B20] Markus H. Kunda Z. (1986). Stability and malleability of the self-concept. J. Pers. Soc. Psychol. 51, 858–866. 10.1037/0022-3514.51.4.858 3783430

[B21] Markus H. Nurius P. (1986). Possible selves. Am. Psychol. 41, 954. 10.1037/0003-066X.41.9.954

[B22] Mehling W. E. Price C. Daubenmier J. J. Acree M. Bartmess E. Stewart A. (2012). The multidimensional assessment of interoceptive awareness (MAIA). PLoS ONE 7, e48230. 10.1371/journal.pone.0048230 23133619 PMC3486814

[B23] Moussally J. M. Grynberg D. Goffinet S. Simon Y. Van der Linden M. (2017). Novel assessment of own and ideal body perception among women: validation of the computer-generated figure rating scale. Cognit. Ther. Res. 41, 632–644. 10.1007/s10608-016-9827-4

[B24] Nakul E. Orlando-Dessaints N. Lenggenhager B. Lopez C. (2020). Measuring perceived self-location in virtual reality. Sci. Rep. 10, 6802. 10.1038/s41598-020-63643-y 32321976 PMC7176655

[B25] Paterson C. Lengacher C. A. Donovan K. A. Kip K. E. Tofthagen C. S. (2016). Body image in younger breast cancer survivors: a systematic review. Cancer Nurs. 39, E39. 10.1097/NCC.0000000000000251 25881807 PMC4607543

[B26] Raimo S. Boccia M. Di Vita A. Cropano M. Guariglia C. Grossi D. . (2021). The body across adulthood: on the relation between interoception and body representations. Front. Neurosci. 15, 586684. 10.3389/fnins.2021.586684 33716641 PMC7943607

[B27] Reed R. G. Weihs K. L. Sbarra D. A. Breen E. C. Irwin M. R. Butler E. A. (2016). Emotional acceptance, inflammation, and sickness symptoms across the first two years following breast cancer diagnosis. Brain Behav. Immun. 56, 165–174. 10.1016/j.bbi.2016.02.018 26916219 PMC4917434

[B28] Savioni L. Triberti S. Durosini I. Sebri V. Pravettoni G. (2022). Cancer patients' participation and commitment to psychological interventions: a scoping review. Psychol. Health 37, 1022–1055. 10.1080/08870446.2021.1916494 33966548

[B29] Sebri V. Durosini I. Mazzoni D. Pravettoni G. (2022). The body after cancer: a qualitative study on breast cancer survivors' body representation. Int. J. Environ. Res. Public Health 19, 12515. 10.3390/ijerph191912515 36231811 PMC9566341

[B30] Sebri V. Triberti S. Pravettoni G. (2020). Injured self: autobiographical memory, self-concept, and mental health risk in breast cancer survivors. Front. Psychol. 11, 607514. 10.3389/fpsyg.2020.607514 33250833 PMC7672015

[B31] Serino S. Pedroli E. Keizer A. Triberti S. Dakanalis A. Pallavicini F. . (2016). Virtual reality body swapping: a tool for modifying the allocentric memory of the body. Cyberpsychol. Behav. Soc. Netw. 19, 127–133. 10.1089/cyber.2015.0229 26506136

[B32] Sherman K. A. Przezdziecki A. Alcorso J. Kilby C. J. Elder E. Boyages J. . (2018). Reducing body image–related distress in women with breast cancer using a structured online writing exercise: results from the my changed body randomized controlled trial. J. Clin. Oncol. 36, 1930–1940. 10.1200/JCO.2017.76.3318 29688834

[B33] Slater M. Pérez Marcos D. Ehrsson H. Sanchez-Vives M. V. (2008). Towards a digital body: the virtual arm illusion. Front. Hum. Neurosci. 2, 6. 10.3389/neuro.09.006.2008 18958207 PMC2572198

[B34] So B. P. H. Lai D. K. H. Cheung D. S. K. Lam W. K. Cheung J. C. W. Wong D. W. C. (2022). Virtual reality-based immersive rehabilitation for cognitive-and behavioral-impairment-related eating disorders: a VREHAB framework scoping review. Int. J. Environ. Res. Public Health 19, 5821. 10.3390/ijerph19105821 35627357 PMC9141870

[B35] Spielberger C. Gorsuch R. Lushene R. Vagg P. Jacobs G. (1983). Manual for the Stait-Trait Anxiety Inventory. Palo Alto, CA: Consulting Psychologists Press.

[B36] Spielberger C. D. (1983). State-Trait Anxiety Inventory for Adults. Washington, DC: APA PsycTests. 10.1037/t06496-000

[B37] Thompson J. K. Heinberg L. J. Altabe M. Tantleff-Dunn S. (1999). Exacting beauty: theory, assessment, and treatment of body image disturbance. Am. Psychol. Assoc. 10.1037/10312-000

[B38] Ventura S. Cebolla A. Herrero R. Baños R. M. (2018). “Qualitative research of an innovative virtual reality embodiment system: the machine to be another,” in Proceedings of the 12th International Conference on Disability Virtual Reality and Associated Technologies.

